# Oxidative Stability and Antioxidant Activity in Canned Eels: Effect of Processing and Filling Medium

**DOI:** 10.3390/foods10040790

**Published:** 2021-04-07

**Authors:** Lucía Gómez-Limia, Nicolás Moya Sanmartín, Javier Carballo, Rubén Domínguez, José M. Lorenzo, Sidonia Martínez

**Affiliations:** 1 Área de Tecnología de los Alimentos, Facultad de Ciencias de Ourense, Universidad de Vigo, 32004 Ourense, Spain; lugomez@uvigo.es (L.G.-L.); Nmoya@alumnos.uvigo.es (N.M.S.); carbatec@uvigo.es (J.C.); jmlorenzo@ceteca.net (J.M.L.); 2 Centro Tecnológico de la Carne de Galicia, Rúa Galicia N_4, Parque Tecnológico de Galicia, San Cibrao das Viñas, 32900 Ourense, Spain; rubendominguez@ceteca.net

**Keywords:** canned eels, filling medium, olive oil, sunflower oil, oxidation, antioxidants, total phenols, vitamin E

## Abstract

The effect of canning and the use of different filling media (sunflower oil, olive oil, and spiced olive oil) on oxidation parameters (acidity, peroxide value (PV), and thiobarbituric acid reactive substances (TBARS) index), antioxidant capacity, and total phenol and vitamin E contents in eels was studied. A preliminary frying treatment caused a decrease in titratable acidity and an increase in TBARS, antioxidant capacity, and vitamin E in the eel muscle. During sterilization, TBARS also increased significantly. The magnitude of the changes depended on the filling medium. Storage also had a significant effect on oxidation parameters in eel muscle and in filling media. After one year of storage, the sunflower oil and canned eels packed in this oil presented higher antioxidant capacity and vitamin E content than olive oil, spiced olive oil, or canned eels packed in these oils. However, the total phenol contents were higher when olive oil or spiced olive oil were used as filling media. Despite the losses, the results show that the canning process and subsequent storage preserved a great part of the antioxidant capacity and vitamin E content of the filling medium, which is of interest to the consumer. Both sunflower oil and olive oil as filling media are of great nutritional interest.

## 1. Introduction

Canned fish are products obtained from various marine species, packed in airtight containers with different filling media, and then sterilized by heat treatment. Canned fish is one of the most popular forms of fish consumption due to its properties of healthy and high nutritional value, storability, availability, and ready-to-eat nature. However, the quality of canned fish can vary with different factors such as type of fish, fresh fish quality, treatment conditions (time, temperature, and container type), filling medium, etc. [1,2]. The type of filling medium and its state strongly affect determine the quality of canned fish. The filling medium influences the nutrient content of the final product and can produce dilution and extraction of some components of the fish muscle [3]. Moreover, during and after heat treatment, complex interactions between components of fish and the filling medium take place, which also affect the characteristics of the canned fish. Different filling media such as brine, olive oil, sunflower oil, soybean oil, and other vegetable oils can be used in the packing of canned fish, with the olive and sunflower oils being the most frequently used.

During processing and storage, fish and filling mediums undergo modifications because of mechanical, thermal, hydrolytic, and oxidative degradations. Particularly, oxidative reactions during heat treatment produce a deterioration of the oil, decreasing its quality and affecting canned fish. Lipid oxidation is very important to the quality of foods, especially of those having highly unsaturated fatty acid contents, and it can cause nutrient losses, unpalatable flavors and odors, shortening of shelf life, and possible production of unhealthy molecules [4,5]. In this regard, the rancidity of the oils has been linked to harmful health effects such as cancer and neurological disorders because of the production of potentially toxic compounds [6].

In addition, fish lipids are rich in long-chain, highly unsaturated fatty acids, which are highly susceptible to oxidation [7,8]. However, oxidation can be minimized in the canned fish by using filling media containing natural antioxidants such as tocopherols or polyphenols [9].

On the other hand, in order to improve the flavor, some spices and aromatic herbs can also be added to the filling medium. These spices and herbs also contribute with their antioxidant and antimicrobial capacities to the nutritional, organoleptic, and healthy characteristics of the final products [10,11,12].

The European eel (*Anguilla anguilla*) is an important fish species in some European and Asian countries despite the fact that the excessive capture of juveniles and the impossibility of captive breeding make its availability limited. The canning process would enable eels to be consumed throughout the year while respecting the regulations aimed at protecting the species.

The European eel is a fish with a high fat content. It has a high content of monounsaturated (51.77%) and polyunsaturated (15.22%) fatty acids. The canning process and filling medium relate to its fatty acid profile [1]. Canning can also cause oxidation during fish processing.

Due to the considerations previously made about the peculiarities of the fish lipids and the effects of the canning process and filling media on the quality of the final product, the aim of this work was the study of the effect of canning and subsequent storage, and of different filling media (sunflower oil, olive oil, and spiced olive oil), on the oxidation processes, antioxidant capacity, and content in some antioxidant compounds such as phenols and vitamin E, both in canned eels and in the filling media.

## 2. Materials and Methods

### 2.1. Sample Preparation

European eels (*Anguilla anguilla*) were obtained from a local market (Mariscos Vivos del Grove, Plaza de Abastos) in Ourense (Galicia, Spain). All of the individual eels used in the study weighed between 200 and 600 g, and they were purchased immediately after capture. They were transferred to the laboratory, where they were eviscerated and frozen stored for a maximum of one month at −20 °C. Some randomly selected samples of the frozen eels, hereafter referred to as “raw eels,” were thawed in a refrigerator at 4 °C for 12 h and processed as control samples.

In order to make canned eels, the fish were thawed in 12% brine at room temperature for 45 min. The eels were cut into slices and, after mixing to ensure product homogeneity, the slices were fried at 190 °C for 2 min in a conventional frying pan using refined sunflower or olive oil as frying media. The fried slices were then cooled (until 30 °C) and placed into 125 mL glass cans (6 or 7 slices in each). The hot filling medium (sunflower oil for eels previously fried in sunflower oil and olive oil or olive oil plus chili and pepper for eels previously fried in olive oil) was then added. The filling medium with olive oil plus chili and pepper will be referred to throughout this paper as “spiced olive oil.” The drained weight of eel and the amount of oil added in each can were 50.00 ± 3.91 g and 34.9 ± 3.4 g, respectively. Next, the cans were vacuum-sealed, sterilized at 118 °C for 30 min (F_0_ = 11), and finally cooled and stored at room temperature.

Eels were sampled raw (control), after each processing step (frying and sterilization treatment), and throughout the storage of the canned product (after 2 and 12 months of storage). The same sampling protocol was followed for the oil samples. Before analysis, the cans were opened and the filling oil was carefully drained off gravimetrically through a 3 mm pore sieve and then filtered for 3 min, thus obtaining the fish and the oil separately.

### 2.2. Measurement of the Acidity, Peroxide Value, and Thiobarbituric Acid Reactive Substances Index in Eels and Oils

The acidity of the eel muscle was determined according to the official method of the Association of Official Agricultural Chemists (AOAC) [13]. The sample (2.5 g) was homogenized with distilled water (25 mL) and left standing for 1 min. Subsequently, the homogenate was filtered, and the filtrate (25 mL) was titrated with NaOH (0.1 N) until pH 8.12. Titratable acidity was expressed as % of lactic acid.

The acidity degree of the oils was assessed according to the procedure described by the European Regulation [14]. Results were expressed as a percentage of oleic acid.

Fat from eel was extracted according to Bligh and Dyer [15] using a mixture of chloroform–methanol (2:1 *v*/*v*) as solvent. The peroxide value (PV) of eel fat and filling oils was determined according to standard methods for the oils analysis proposed by the American Oil Chemists' Society (AOCS) [16] and was expressed as meq. O_2_/kg of the sample (fat).

The thiobarbituric acid reactive substances (TBARS) index was determined according to the Kirk and Sawyer [17] method. Malondialdehyde (MDA) is the main product resulting from the degradation of hydroperoxides generated by lipid oxidation, and the reaction between MDA and the thiobarbituric acid (TBA) generates a pink-red compound measurable by spectrophotometry using wavelengths between 532–535 nm. The results were expressed as mg malondialdehyde/kg of sample.

### 2.3. Determination of Antioxidant Activity

For the determination of antioxidant activity, eel or oil extracts were prepared in ethyl acetate (eel muscle: 0.5 g/mL; oils: 0.1 g/mL). The mixtures were homogenized for 1 min (IKA T25 digital Ultra-Turrax, Wilmington, EEUU), sonicated for 15 min (Branson 3510 Ultrasonic Cleaner; Branson Ultrasonics Corporation, Danbury, CT, USA), and subsequently centrifuged at 14,000× *g* for 10 min in an Eppendorf centrifuge 5804R (Eppendorf AG, Hamburg, Germany). The supernatants were collected, filtered, and finally stored at −30 °C until analysis.

The antioxidant activity was determined by assessing the 2,2-diphenyl-1-picryl-hydrazyl-hydrate (DPPH) free radical scavenging activity of the eel or oil extracts according to the method described by Brand-Williams et al. [18], with some modifications. When the DPPH radical reacts with an antioxidant compound, it is reduced and changes its color. Two mL of different extract concentrations (muscle of eel: 100, 50 and 12 mg/mL; oils: 25, 10 and 5 mg/mL) were mixed with 0.5 mL of a DPPH solution in ethyl acetate (0.2 mmol/L, *v*/*v*). The reaction mixture was maintained in the dark for 60 min. The color changes were read as absorbance at 517 nm in a Shimadzu UV-1800 spectrophotometer (Shimadzu Corp., Kyoto, Japan) against a control solution prepared by mixing pure ethyl acetate (2 mL) and the DPPH radical solution (0.5 mL).

The antioxidant activity was expressed in terms of IC_50_ DPPH. The IC_50_ DPPH is defined as the extract concentration (mg/mL) required to decrease the initial DPPH concentration by 50%. This value was calculated by linear regression analysis of the dose–response curve obtained by plotting the radical scavenging activity against extract concentration [19]. To calculate the IC_50_ DPPH different percentages of inhibition were calculated as follows:

% Inhibition = [(A_control_ − A_sample_)/A_control_] × 100,

where A_control_ is the absorbance of the blank at 0 time, and A_sample_ is the absorbance of the sample.

### 2.4. Determination of Total Phenolic Content

Samples (3 g muscle or oil) were mixed with 10 mL aqueous methanol (80/20 *v*/*v*). After extraction following the previously described procedure, total phenolic content was determined by the Folin–Ciocalteu colorimetric method assay described by Singleton and Rossi with some modifications [20]. Aliquots of 0.5 mL of extract were mixed with 2.5 mL of Folin–Ciocalteu reagent and 2 mL of NaCO_3_ (7.5%; *p*/*v*). The mixture was heated at 45 °C for 15 min in a water bath in darkness and left standing for 30 min before measurement of the absorbance at 765 nm. The total phenolic content was expressed as gallic acid equivalents (GAE)/100 g of sample, after interpolating the absorbance values in a standard curve obtained using different concentrations of gallic acid (5–500 mg/L).

### 2.5. Determination of Vitamin E Content

The vitamin E content was determined by HPLC techniques, using a ThermoFinnigan (Silicon Valley, CA, USA) chromatograph equipped with a UV/VISIBLE photodiode array detector Spectrasystem UV6000LP.

Two and a half g of eel muscle were saponified by mixing with 4 mL of 50% KOH and 6 mL of ethanol. The mix was then left for 30 min in a water bath at 80 °C under dark conditions. To avoid deterioration of vitamin E during saponification, 0.25 g of ascorbic acid was added to each sample. Then, the mix was cooled and after 10 mL of hexane and 5 mL of distilled water were added, it was homogenized in a vortex and centrifuged at 4200× *g* for 5 min. Next, the supernatant (4 mL) was collected and dried in a stream of N_2_. The dried residue was then reconstituted with 1.5 mL of HPLC quality methanol and filtered into a vial. The vitamin E was identified and quantified using a C18 reversed-phase column of 5 μm particle size, diameter 4.6 mm, and length 25 cm (Ultrasphere 5–ODS, Beckman, Fullerton, CA, USA). The detector wavelength was 294 nm. The chromatographic conditions were 1 mL/min flow for 25 min with a mixture of 96:4 (Methanol:MilliQ water). The standards were prepared by successive dissolution of α-tocopherol in methanol and saponification of the mixture as for the samples.

For the determination of vitamin E in oils, 1:10 dilutions of oil in isopropanol were used. The same HPLC system with a fluorescence (FLD-3100) detector was used. The detector was set at an excitation wavelength of 290 nm and an emission wavelength of 330 nm. A normal phase silica column (SunFire™ Prep Silica, 4.6 mm ID × 250 mm, 5 µm particle size, Waters, Milford, MA, USA) was used. The column oven was thermostated at 30 °C. A total of 10 µL of each sample and standard were injected. The chromatographic conditions used were 1 mL/min flow of hexane:isopropanol (98:2) during 15 min. Standards were prepared by successively dissolving α-tocopherol in isopropanol.

### 2.6. Statistical Analysis

All analyses were carried out at least in triplicate. The data were examined by analysis of variance (ANOVA), and the least-squares test (LSD) was used (*p* < 0.05) to compare the mean values. The tests were implemented using the Statistica software, version 7.1 (Statsoft© Inc., Tulsa, OK, USA). Correlations between the different parameters analyzed were determined by multiple regressions, with confidence intervals of 95% (*p* < 0.05), 99% (*p* < 0.01) and 99.9% (*p* < 0.001).

## 3. Results and Discussion

### 3.1. Effect of Processing and Filling Medium on Acidity

Acidity has important effects on muscle quality. Its changes can be used as an indicator of postmortem transformation of glycogen into lactic acid and of the degradation of muscles during storage.

**Figure 1:**
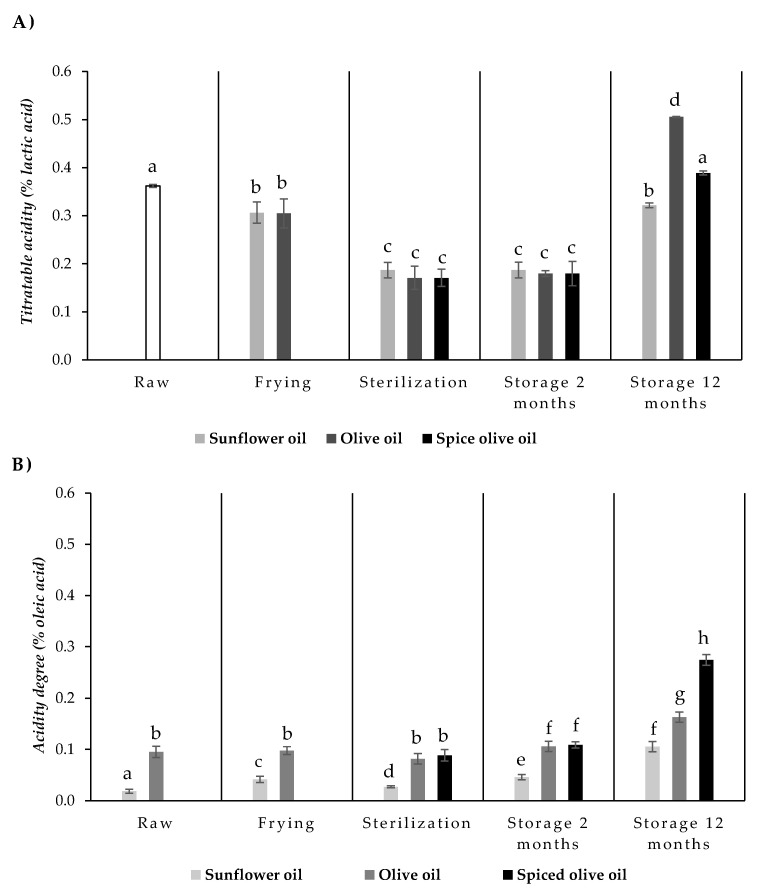
Titratable acidity (% lactic acid) of eels (**A**) and acidity degree (% oleic acid) of filling oils (**B**) throughout the different steps of the canning process (raw, after frying, and after sterilization process) and after 2 and 12 months of storage. Plotted values are mean of at least three determinations ± standard deviation. ^a–h^ Mean values with different letters are significantly different (*p* < 0.05).

The acidity in the eel muscle was calculated as titratable acidity (% lactic acid). Values of titratable acidity values of raw eels and eels packed in sunflower oil, olive oil, or spiced olive oil, at each step of the canning process and after room storage (for 2 and 12 months) are shown in Figure 1A. Titratable acidity (0.36% lactic acid in raw eels) decreased after frying and sterilization processes. This can be due to the loss of organic acids to the frying oils and the destruction of some heat-labile acids. There were no significant differences associated with the type of oil (sunflower or olive) used in the processes. The lower acidity values in canned fish compared to the raw samples could be due to the formation and accumulation of some dibasic amino acid and volatile basic nitrogenous compounds due to breakdown and proteolysis during heat treatment [21].

During the first two months of storage, titratable acidity remained stable in all canned eels. However, the values increased after 12 months of storage. This increase may be due to the release of organic acids from the food matrix or to the formation of new acid compounds during storage. Titratable acidity values at the end of the storage were significantly (*p* < 0.05) affected by the filling medium, and the highest values were observed in canned eels packed in olive oil (0.51% lactic acid).

In order to check the variations in the acidity of the oils, this parameter was measured in raw sunflower and olive oils, and also after frying, sterilization treatment, and storage during 2 and 12 months (Figure 1B). The acidity degree of the oils depends on their content of free fatty acids. It is expressed as a percentage of oleic acid, the main fatty acid present in oils, and allows the evaluation of the behavior of the oil during heat treatment and storage. The acidity of the raw oils was 0.018 % oleic acid and 0.101% oleic acid for the sunflower and olive oils, respectively, and the differences were significant (*p* < 0.05). Usually, the susceptibility to oxidation is higher in the oils having higher initial acidity values. The acidity degree of oils is a very important parameter and depends on the intensity of the refining process carried out since during this process the free fatty acids were partially removed using different chemical or physical processes. In the case of olive oil, the acidity degree is used as a classification criterion that allows distinguishing among refined (<0.3%), extra virgin (not more than 0.8%), virgin (<2%), and olive oil (<3.3%); the oil is considered unsuitable for consumption when acidity degree is >2% [22].

Frying resulted in a significant (*p* < 0.05) increase in the acidity degree value in sunflower oil (to 0.04% oleic acid); however, no significant changes were observed in the olive oil (0.09% oleic acid) after this process. During frying, the oils undergo three different reactions—hydrolysis, oxidation, and thermal degradation. These reactions do not occur to the same extent in all vegetable oils [23]. The hydrolysis also leads to an increase in acidity, with further formation of methyl ketones and lactones that cause unpleasant smells and tastes. The high temperatures can significantly deteriorate the oils, especially if they are highly unsaturated since oxidation products are formed.

The sterilization process caused a decrease in the acidity degree of sunflower oil (0.026% oleic acid) with respect to the fried oil; however, no significant changes were observed in olive oil and spiced olive oil (0.08% and 0.09% oleic acid, respectively), compared to fresh olive oil. During the frying and sterilization processes, the composition of the fried or packed food and that of the oil/fat used as frying or filling medium change continuously, mainly due to the alteration of the oil, but also due to changes in the food, and reactions of interaction between oil and food. A higher degree of acidity indicating a higher release of fatty acids from triglycerides means a greater degree of deterioration due to the heat damage. Additionally, the decrease in the acidity values seems to be the result of free fatty acid degradation via oxidation processes.

Usually, sunflower oil has a higher content of α-tocopherols, but in our case, these did not seem to protect the oil against oxidation, probably due to the higher content of easily oxidizable linoleic acid [24]. In the case of olive oil, heat treatments caused fewer changes in the acidity degree than in sunflower oil. This trend has also been pointed out in other studies since olive oil is rich in antioxidant compounds such as phenolic compounds and tocopherols [25,26]. On the other hand, olive oil has a higher concentration of monounsaturated fatty acids, such as oleic acid, which are more resistant to degradation than polyunsaturated fatty acids, such as linoleic acid. Sunflower oil had greater amounts of polyunsaturated fatty acids (PUFAs); thus, taking into account that oxidation susceptibility is correlated exponentially with the number of unsaturation of fatty acids [5], this could partially explain our results.

Storage significantly (*p* < 0.005) increased the acidity degree in the three oils studied. This increase was higher in the spicy olive oil after 12 months of storage (to 0.27% oleic acid). During storage, many compounds migrate from fish to filling medium and vice versa. The balance established depends on the type of fish and previous treatments of it, the type of filling medium, the canning technology, and storage conditions. Spices and condiments can sometimes act as pro-oxidants in canned food [10].

In the present study, the spices used in the preparation of spiced olive oil, pepper, and chili, can also undergo an oxidation process during processing and storage, which may contribute to the increase in the degree of acidity of the spiced olive oil. Additionally, during storage, the spices could release some components and these can migrate from spices to the oil and increase acidity to the filling medium [27].

### 3.2. Effect of Processing and Filling Medium on Peroxide and TBARS Values

The peroxide value is often used to measure the deterioration of oils. This value is related to treatments and storage conditions (oxygen, light, temperature, metals, enzymes, presence of antioxidants or pro-oxidants, fatty acid composition, the use of oxygen-permeable packages, etc.) [28]. It allows estimating the degree of oxidation of fatty acids. Thermal treatment and high storage temperatures can increase the susceptibility of unsaturated fatty acids toward oxidation resulting in losses of these compounds. Dimerization and polymerization are important reactions in the thermal oxidation in oil [29]. The heat temperature and time, filling oil or antioxidant contents, affect the hydrolysis, oxidation, and polymerization of the fat. The degree of fat oxidation in fish can also be determined by assessing the thiobarbituric acid reactive substances (TBARS) values that evaluate the formation of secondary oxidation products, especially malonaldehyde (MDA) and its further transformation and/or reaction with the processed products. Lipid oxidation consists of three stages—initiation, propagation, and termination. The peroxides that are normally formed as primary products can subsequently undergo scission to form secondary oxidation products such as carbonyls, alcohols, hydrocarbons, and furans through different reactions such as cyclization, hydrogen abstraction, addition reaction, recombination, scission, and polymerization [30]. The aldehydes, such as hexanal malondialdehyde (MDA), are one of the most abundant products found [31].

**Figure 2:**
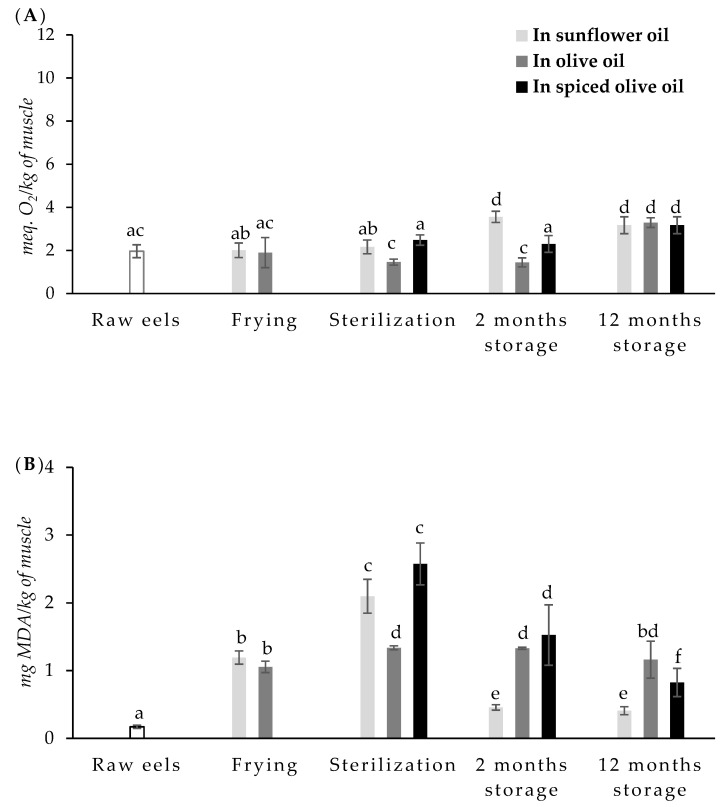
Peroxide values (PVs) (meq. O_2_/kg of the sample) (**A**) and thiobarbituric acid reactive substances (TBARS) values (mg malonaldehyde/kg of the sample) (**B**) of raw and canned European eel in each step during the canning process and at 2 and 12 months of stored. ^a–f^ Mean values with different letters are significantly different (*p* < 0.05).

PV (expressed as meq. O_2_/kg of the sample) and TBARS (mg malonaldehyde/kg of the sample) of raw and canned eels sampled at each stage of the canning process (after frying, after sterilization process, and after room storage for 2 and 12 months) are shown in Figure 2.

Peroxide values of raw eels ranged from 1.71 to 2.30 meq. O_2_/kg. These data were consistent with those reported by Selmi et al. [32] in fresh tuna (2.50 meq. O_2_/kg) but lower than the values observed by these same authors in sardine (8.14 meq. O_2_/kg). In that study, fresh sardine showed higher PUFAs content and a lower monounsaturated fatty acids (MUFAs) fraction than fresh tuna. The European eel has a high content of monounsaturated fatty acids (51.77%), followed by saturated fatty acids (SFAs) (32%) and polyunsaturated fatty acids (PUFAs) (15.22%) [1].

It has been reported that the maximum level of TBARS values indicating good quality of the fresh fish during storage is 1–2 mg MDA/kg [33]. In the present work, the TBARS values observed in raw eels were very lower (0.17 mg MDA/kg) than those indicated figures. Ehsani and Jasour [34] found even lower values in rainbow trout fillets (0.06 mg MDA/kg) such as Naseri et al. [35] in silver carp muscle (0.01 mg MDA/kg). However, Kong et al. [36] reported higher TBARS values in pink salmon muscle (4.59 mg MDA/kg). These differences could be due to different concentrations of fat in the fish muscle [1] and different storage times and conditions before analysis.

Frying did not have a significant effect on PV when sunflower oil or olive oils were used as frying media. Peroxides are unstable under frying conditions, and the use of oil for frying does not lead to substantial increases in peroxide values because peroxides decompose spontaneously above 150 °C to form secondary oxidation products [37]. However, frying caused a significant increase in TBARS values. No significant differences were observed between TBARS values of the samples fried in sunflower oil (1.19 mg MDA/kg) and the samples fried in olive oil (1.06 mg MDA/kg).

The sterilization process did not increase PVs either. The PV levels were lower than those observed by El-Shehawy and Farag [33] in canned tuna, canned sardine, and canned mackerel in different filling media.

During the treatment of sterilization, TBARS values increased significantly. In this case, the increase was higher in canned eels packed in sunflower oil (2.10 mg MDA/kg) or in spiced olive oil (2.57 mg MDA/kg) than in canned eels packed in olive oil (1.34 mg MDA/kg). Selmi et al. [32] reported that cooking increased PV in sardine, but canning did not cause significant changes. In this same line, Uriarte-Montoya et al. [38] indicated that the canning did not have a significant effect on the oxidation in Pacific sardine (*Sardinops sagax caerulea*).

The high treatment temperatures or long treatment times cause an increase in secondary oxidation products, such as malonaldehyde [39]. Naseri et al. [35] did not observe significant differences between the TBARS values of raw silver carp and precooked samples before the canning process. However, they observed a significant increase in TBARS values during sterilization (canning) in silver carp packed in sunflower oil and soybean oil, but not in canned silver carp packed in olive oil. Kong et al. [36] observed a slight increase during the first 10 min of heating, followed by a significant decrease in TBARS values as heating progressed.

Medina et al. [40] reported significant differences between TBARS values in canned tuna when different filling media were used. TBARS values were higher in canned tuna muscle using brine as the filling medium than in canned tuna packed in extra virgin olive oil. These authors suggested that the antioxidant activity of phenolic compounds of olive oil could be responsible for these differences. Bilgin and Gençcelep [41] also found significant differences among TBARS values of fish canned in different filling media.

Canned storage is normally necessary to produce satisfactory textural and optimal palatability of canned fish. Although the shelf life of a canned fish is between one and five years, storage time can be variable. In this study, the effect of short storage (two months) and longer storage (12 months) was evaluated. Storage had a significant effect on PV in canned eels. The PV increased significantly after two months of storage in canned eels packed in sunflower oil, and then it was stable until 12 months of storage. In the case of canned eels packed in olive oil or in spiced olive oil, PV remained stable during the first two months of storage but significantly (*p* < 0.05) increased after 12 months of storage. No significant differences were observed between the three types of canned eels at 12 months storage. Selmi et al. [32] reported an increase in PV levels after six months of storage in canned tuna. The high temperatures during processing can accelerate oxidation, but filling medium can dissolve oxidation products, or these can be transformed into secondary oxidation products, reducing their concentration in the fish [29]. It has been pointed out that at high temperatures, the initial hydroperoxides formed exist only briefly and will be quickly decomposed into various volatile and nonvolatile products [42]. Moreover, lipid oxidation products can interact with proteins, which could explain in some cases a decrease in primary and secondary oxidation products [43]. Heat and storage can also prompt lipid hydrolysis. The free fatty acids, which appear as a result of hydrolysis, can further undergo oxidation reactions. A direct correlation between lipolysis and lipid oxidation of seafood products has been reported [44]. These free fatty are more susceptible to undergo oxidative reactions in order to form primary and secondary oxidative products [45].

In addition, the filling medium used can determine the peroxide value of the canned fish. Leung et al. [46] found in salmon subjected to different treatments that frying, baking, or using old oil led to an increase in peroxide values, while cooking did not produce significant changes. Al-Saghir et al. [47], in salmon and trout, reported that during frying, salmon fried in olive oil or corn oil increased its peroxide value, but the increase was much greater in baked trout and fried trout in sunflower oil. However, Talab [43] observed that different treatments produced a decrease in peroxide values in carps. The variation in the hydroperoxides formed in the fried and canned samples can be attributed to the influence of the frying or filling medium. In order to evaluate the effect of the filling medium, the PV in the oils was also determined.

Storage during two months caused a decrease in TBARS values in canned eels packed in sunflower oil and canned eels packed in spiced olive oil; this decrease was more important in canned eels packed in sunflower oil. TBARS values continued to decrease in canned eels packed in spiced olive oil until 12 months of storage. In canned eels packed in olive oil or sunflower oil, TBARS values did not undergo significant changes throughout the remaining 10 months of storage. The decrease of the TBARS values can be due to the disappearance of the malondialdehyde through reactions with amines, nucleosides, nucleic acids, amino-containing phospholipids, proteins, or other aldehydes that are also by-products of lipid oxidation [41]. The further oxidation of primary products and the formation of carboxylic acids and other compounds that are not reactive to the 2-thiobarbituric acid can be also the cause of the decrease of the TBARS values during storage [5,48]. The changes in TBARS values may also be explained by the different interactions between fish muscle and filling medium, by the diluting effect of the filling medium on secondary oxidation products, and/or by the effect of the thermal treatment.

**Figure 3:**
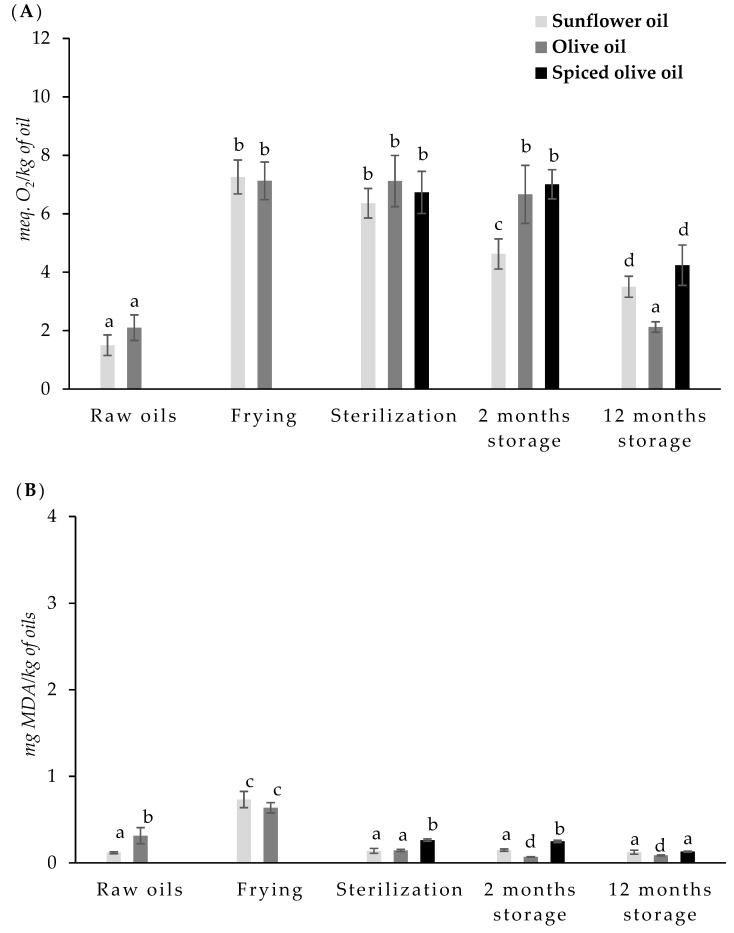
Peroxide values (PVs) (meq. O_2_/kg of the sample) (**A**) and thiobarbituric acid reactive substances (TBARS) values (mg malonaldehyde/kg of the sample) (**B**) of oils in each step during the canning process and at 2 and 12 months of stored. ^a–d^- Mean values with different letters are significantly different (*p* < 0.05).

PV (meq. O_2_/kg of the sample) and TBARS (mg MDA/kg of the sample) of oils sampled at each stage of the canning process (raw, after frying, after sterilization process, and after room storage for 2 and 12 months) are shown in Figure 3.

Peroxide values in raw oils were 1.50 and 2.49 meq. O_2_/kg in sunflower oil and olive oil, respectively. The raw oils presented TBARS values of 0.12 mg MDA/kg and 0.31 mg MDA/kg for sunflower and olive oil, respectively.

Frying caused a significant increase in PV with respect to crude oils (7.26 and 7.13 meq. O_2_/kg of oil in sunflower and olive oils, respectively). However, the sterilization process did not cause significant changes in PVs of the oils.

TBARS values also increased after frying, with the increase being higher in sunflower oil (0.73 mg MDA/kg) than in olive oil (0.64 mg MDA/kg). However, sterilization treatment resulted in a significant decrease in the TBARS index in all oils. In this case, this decrease was higher in sunflower oil and in olive oil than in spiced olive oil.

Storage produced a decrease in PV in sunflower oil. In the case of olive oil or spiced olive oil, no significant differences were observed after two months of storage. However, after 12 months of storage, a decrease in PV was also observed in olive and spiced olive oil. The lowest PVs were observed in olive oil after 12 months of storage.

No significant changes in TBARS values of sunflower oil were observed during storage. In the case of olive oil, TBARS values decreased after two months of storage. On the other hand, in the spicy olive oil, no changes were observed after two months of storage, but there was a decrease after one year of storage in relation to the values obtained after sterilization.

Alhibshi et al. [49] pointed out that when the peroxide values are between 30 and 40 meq. O_2_/kg the rancid flavor is clearly noticeable. In the samples under study, these indicated values were not observed. Different studies are carried out on the evolution of the peroxide value in oils after heat treatment. Naz et al. [50] found that the peroxide value increased in olive oil during frying, and this increase was proportional to the treatment time. Alajtal et al. [51] also observed an increase in the peroxide value in sunflower and olive oils after frying. The increase in the peroxide value during frying and sterilization is due to oxidation processes. A possible explanation for the peroxide values measured during the frying and sterilization process is that the rate of degradation is similar to the rate of formation, increasing TBARS values.

The subsequent decrease during storage occurs when the peroxides are transformed into other chemical compounds, such as aliphatic carbonyls [5,28]. Since the peroxides formed are not stable compounds, the peroxide value is not always correlated with the degree of oxidation of oils [5].

The results seem to be related to the fatty acid composition of oils. When oils are exposed to high temperatures, the polyunsaturated fatty acids are affected, and the oils with a high n-3 PUFA level experience a greater effect on TBARS values than oils with a low n-3 PUFA level. Heat treatments cause the oil to experience a series of chemical reactions such as oxidation, hydrolysis, and polymerization [52].

Caponio et al. [53] evaluated the effect of the replacement of refined oils with extra virgin olive oil in bakery products rich in fat. They observed that the evolution of the oxidation levels in the analyzed samples during storage was related to the type of oil used. The use of extra virgin olive oil led to significantly lower values of hydroperoxides, ultraviolet absorption constants, triacylglycerol, oligopolymers, and oxidized triacylglycerols.

### 3.3. Effect of Processing and Filling Medium on Antioxidant Capacity

Fish tissues can contain different antioxidant components such as enzymes, carotenoids, vitamin E, vitamin C, peptides, amino acids, and phenolic compounds that contribute to their antioxidant defense mechanism. These antioxidants can inhibit the initiation and propagation steps of lipid oxidation. They act acting as free radical scavengers and metal ion chelators. They can have multiple effects, and their mechanisms of action are therefore frequently difficult to interpret. These components are part of the cell plasma, mitochondria, and cell membranes [54]. However, these endogenous antioxidants are consumed sequentially after the death of the fish. Moreover, in the case of canned fish, some of these antioxidant components present in the raw fish can be reduced during processing. In canned fish, the oxidative stability of fish lipids is variable depending on the antioxidant content and stability of the filling medium. In the case of oils, their stability is particularly related to the content of different components, such as polyphenols and tocopherols, which can act as radical scavengers. However, in the case of refined oils, some antioxidant components are lost during the refining operations. Additionally, spices and herbs have been used as a natural source of antioxidant and antimicrobial compounds [11,12].

The DPPH radical is commonly used as substrate in estimating antioxidant activity because of the ability of the antioxidant compounds to reducing the DPPH free radical.

The DPPH radical scavenging activity (expressed in % inhibition of the DPPH radical) of different extracts of muscle eels (100, 50, and 12 mg/mL), both raw and after each step of processing and storage, and canned in different filling media, is shown in Supplementary Materials (Figure S1).

The inhibition at 50% (IC_50_) values are shown in Table 1.

The antioxidant activity of the muscle eel and oil extracts is concentration dependent, and lower IC_50_ values indicate better protective action. The antioxidant capacity of raw eels was low (IC_50_ = 107.05 mg/mL). At the concentration of 100 mg/mL, the percentage of inhibition was 40.48%. The antioxidant capacity of plants has been extensively studied. However, research on the antioxidants from animal sources is limited so far.

In the case of fish, the few existing studies that have been carried out mainly focused on protein hydrolysates of fish. On the other hand, the antioxidant capacity can vary significantly between different fish particularly due to factors such as diet and environmental conditions [55]. A high percentage of fat in some fish, such as eel, can imply the presence of high content of antioxidant compounds. In addition, endogenous antioxidants are sequentially degraded after the death of the fish [56], and the rate of degradation depends on the packaging method and on the storage temperature and time. Therefore, the storage conditions prior to canning have a significant influence on the antioxidant compounds in raw fish and on the final content of oxidation products.

Frying caused a significant increase in antioxidant capacity probably due to the incorporation of some components of the oil into the muscle. In addition, it has been pointed out that the Maillard reaction products and the peptides generated during frying have antioxidant activities [57]. The increase was similar in eels fried in sunflower oil (IC_50_ = 25.24 mg/mL) and eels fried in olive oil (IC_50_ = 28.39 mg/mL). At the highest concentration of the extract (100 mg/mL), the percent of inhibition was 87.9 and 93.6% in eels fried in sunflower oil and olive oil, respectively.

The sterilization process caused a loss of antioxidant capacity in the eel muscle, although it was only significant in eels packed in sunflower oil. These losses can be due to the leaching of soluble components, such as vitamins and proteins, into filling medium and thermal damage during heat treatments [58]. Losses of antioxidant capacity during storage were low. A significant decrease was only observed after twelve months of storage in eels packed in olive oil and spiced olive oil.

The filling media influenced the nutritional quality and antioxidant capacity of canned fish. Medina et al. [40], in canned tuna, found protective effects against lipid oxidation of the extra virgin olive oil rich in phenols and also of the soybean oil rich in tocopherols. Medina et al. [59] investigated the ability of polyphenols extracted from extra virgin olive oil to inhibit lipid oxidation in canned tuna. They observed that a high concentration (400 ppm) of these polyphenols was an effective antioxidant. However, a low concentration (100 ppm) promoted hydroperoxide formation and decomposition. After one year of storage, canned eels packed in sunflower oil presented higher antioxidant capacity than canned eels packed in olive oil or spiced olive oil. Mohan et al. [2] reported that canned tuna packed in sunflower oil as the filling medium offered higher protection compared to other oils such as groundnut oil and coconut oil, possibly due to its protective antioxidant activity. These results can be explained by a high content of natural antioxidants such as tocopherols in sunflower oil.

The DPPH radical scavenging activity of different extracts of oils (25, 10, and 5 mg/mL) is shown in Supplementary Materials (Figure S2).

Concentration-dependent scavenging activity was also found for the studied oils. The resulting DPPH inhibition percentages at the highest concentration (25 mg/mL) were 87.8 and 78.2 % in the raw sunflower and olive oils, respectively. The IC_50_ values were 7.88 and 13.09 mg/mL in raw sunflower and olive oils, respectively. The higher antioxidant activity of the raw sunflower oil can be explained by its richness in tocopherols.

Antioxidant capacity remained stable during frying. No significant differences were detected between the antioxidant capacity of the raw and sterilized oils, except in the case of spiced olive oil. In this case, antioxidant capacity decreased during the sterilization process. As noted above, spices and condiments can sometimes act as pro-oxidants in canned food [10].

In the case of sunflower oil and olive oil, the antioxidant capacity decreased during storage, while in spiced olive oil it remained constant.

### 3.4. Effect of Processing and Filling Medium on Vitamin E Content

Vitamin E is a common term designing tocopherols and tocotrienols. Vitamin E can be found in eight chemical forms (α, β, γ, δ-tocopherol and α, β, γ, δ-tocotrienol) having different levels of biological activity. α-Tocopherol is the predominant form of vitamin E in fish muscle, mainly in marine fish. It has the highest bioavailability and is the most important lipid-soluble antioxidant.

**Figure 4:**
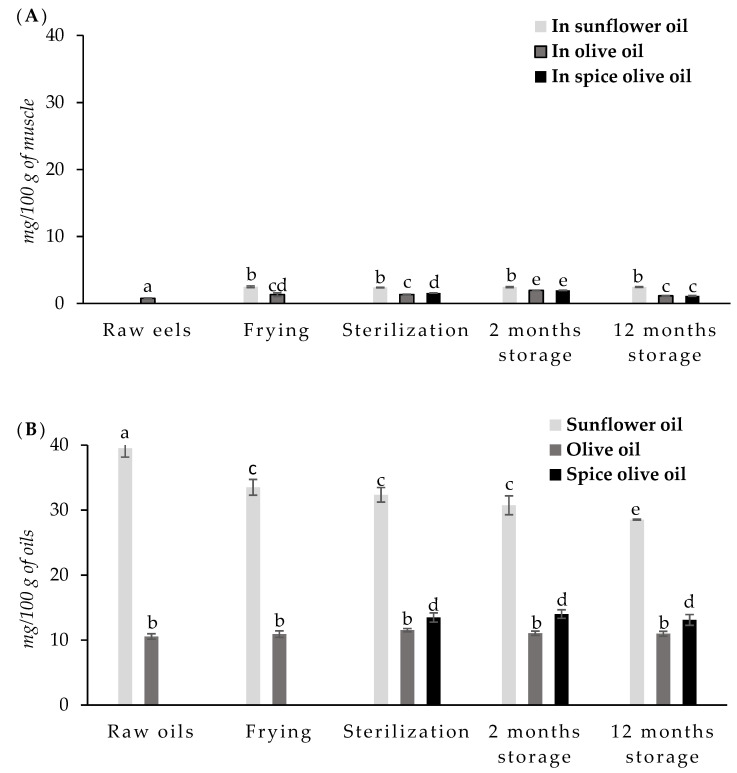
Vitamin E (mg/100 g) of European eel (**A**) and filling oils (**B**) after each step during the canning process and after 2 and 12 months of subsequent storage. ^a–e^ Mean values with different letters are significantly different (*p* < 0.05).

Due to this reason, the α-tocopherol content was determined in the eel muscle after each treatment, and results are shown in Figure 4A.

The α-tocopherol content ranged from 0.69 to 0.88 mg/100 g in raw eels. The vitamin E content in fish can differ widely between species and it is also affected by the season, genetic differences, tocopherols in feed, maturation stage, or storage conditions [4,60]. Additionally, vitamin E content in fish seems to be directly related to lipid content and n-3-PUFA content [4].

The vitamin E content of fried samples increased significantly (*p* < 0.05) when compared to the raw eels. This is probably due to the enrichment from the oil in which eels were fried. The increase was higher in eels fried in sunflower oil than in eels fried in olive oil.

Sterilization process did not have a significant effect on the vitamin E content of fish, even though canned eels packed in spiced olive oil showed higher average values than the fried eels. Ersoy and Özeren [61] reported a significant increase in tocopherol content in African catfish after different treatments such as baking, grilling, microwaving, and frying. Merdzhanova et al. [60] also found a significant increase in tocopherol content after frying in Black Sea horse mackerel.

Vitamin E is sensitive to oxygen, light, and temperature. However, fried and sterilized eels can be enriched in vitamin E due to uptake from the oil and the absorbed quantity depends on the type of oil used in these operations. Sunflower oil has a higher content of vitamin E than olive oil; hence, the highest values in eels fried and packed in sunflower oil. Chilli and pepper can also have a high content of vitamin E [62], which could explain the higher vitamin E content of the samples packed in spiced olive oil.

Vitamin E content remained higher and stable during storage in canned eels packed in sunflower oil. This higher vitamin E content may be related to the higher antioxidant capacity and lower TBARS values of eels packed in sunflower oil at 12 months of storage. However, vitamin E increased during the first two months of storage and then decreased in canned eels packed in olive oil and spiced olive oil. Losses in vitamin E content during storage appear to be due to degradation processes enhanced by factors that influence this degradation such as heat temperatures, time, and exposure to light and oxidative conditions. However, vitamin E is quite stable if foods are adequately protected from conditions favoring lipid oxidation.

Medina et al. [40] reported that there is an inhibition of peroxide decomposition in canned tuna due to polyphenols and tocopherols of filling oil (extra virgin olive oil and refined olive oil). However, they also reported that the protective effect of the polyphenols was higher than that of tocopherols.

The amounts of vitamin E absorbed by fish during frying and/or canning processes depends on the type of oil used [63], and on the number of antioxidants present in the oil. With the purpose of verifying this, the α-tocopherol content was determined in the oils during the frying and canning. Results are summarized in Figure 4B.

Sunflower oil showed a high content of vitamin E (39.51 mg/100 mL) in the present study. These values agree with the concentration found in sunflower oil by Ortiz et al. [64] (35.9 mg/100 mg). Olive oil presented lower content (10.56 mg/100 mL). High variability in the vitamin E content was observed in vegetable oils, depending on different factors such as botanic species, edaphoclimatic environmental conditions, agronomic practices, and extraction procedures [64].

Vitamin E levels significantly decreased in sunflower oil during frying, coinciding with the increase of vitamin E content in the fried eel muscle. Thermal degradation during heating, however, should also contribute to this decrease. No significant changes were observed in the olive oil during the heating process. Quiles et al. [64] reported that the decrease in tocopherol with the frying time depends on the type of oil and time of frying. They reported that olive oils are more stable than sunflower oil during frying processes.

The sterilization process did not cause significant changes in the vitamin E content of the sunflower oil or olive oil. However, this treatment caused an increase in spiced olive oil. Spices such as pepper and chili can contain significant quantities of fat-soluble vitamins, such as tocopherols (mainly vitamin E) [65], which can be dissolved in the oil during the sterilization treatment, thus increasing their concentrations in the oil.

Storage did not modify vitamin E content in olive oil or spiced olive oil; however, it slightly but significantly decreased the vitamin E content in sunflower oil.

### 3.5. Effect of Processing and Filling Medium on Total Phenolic Content

Vegetable oils also contain other natural antioxidants such as phenolic compounds that strongly contribute to their antioxidant capacity. The Folin–Ciocalteau assay is often used to determine the total phenolic content of food [66,67,68].

The total phenol contents were also quantified in fresh fish and raw oils, and in both fish and oils after each stage of processing and storage. Phenols were not detected in either the eels or the sunflower oil. Phenolic compounds can be completely destroyed during the refining process of the oils [69]. Matthaus and Spener [70] reported that carotenoids and phenolic compounds are removed almost totally, while vitamin E-active compounds and phytosterols are reduced by about 10 to 40%.

On the other hand, the phenols present in animal tissues come mainly from food.

**Figure 5:**
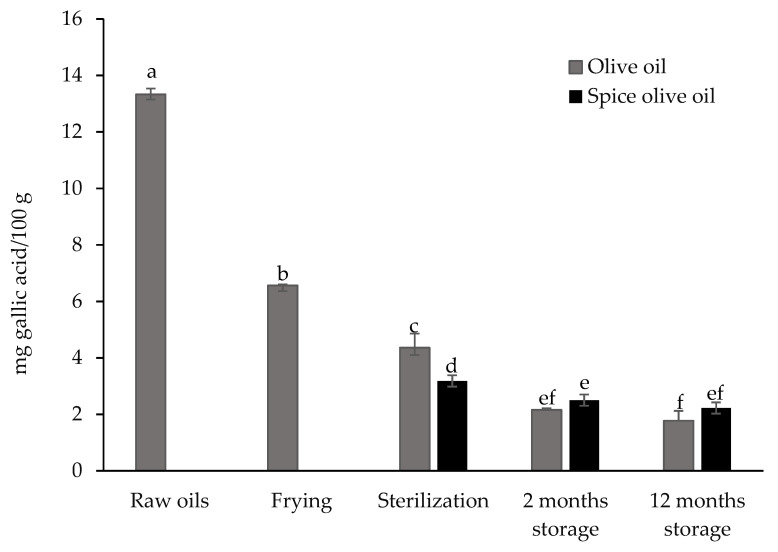
Total phenols (mg gallic acid/100 g) of olive oil and spiced olive oil after each step during the canning process and after 2 and 12 months of subsequent storage. ^a–f^ Mean values with different letters are significantly different (*p* < 0.05).

Total phenolic contents in olive oil and spiced olive oil are shown in Figure 5.

The number of total phenol compounds in olive oil was 13.33 mg GAE/100 g.

According to the literature, the total phenol content in vegetable oils can vary within a wide range depending on the cultivar, environmental edaphoclimatic conditions, cultural practices, and ripening stage of the olives [67,71]. The refining process of virgin olive oil can eliminate practically all the phenolic compounds. In the present study, the olive oil used was a mix of refined olive oils and virgin olive oils, and therefore, phenolic compounds are found.

The frying and sterilization processes caused high losses of phenolic compounds. During sterilization, the highest losses occurred in spiced olive oil. These losses could be due to thermal oxidation reactions, hydrolysis, and polymerization, or to covalent linking between oxidized phenols and proteins or amino acids [25,72]. On the other hand, it has been pointed out that when vegetables are present during heating treatment, they also contribute to the leaching of substances that are released from disrupted cell walls and subcellular compartments [68].

Baiano et al. [73] reported that the phenolic concentration was higher in the extract of the unflavored olive oil than in olive oil flavored with lemons, hot pepper, oregano, rosemary, and garlic. They pointed out that this could be explained based on interactions between the olive oil and the flavoring agents during the extraction phase, which are responsible for the formation of bonds between phenolics and components of fruits, spices, and herbs. This could explain the lower content of phenols detected in the spiced oil in the present work.

A decrease in the total phenolic content was also observed after the first two months of storage in olive oil or spiced olive oil, but from this point, the contents remained stable until 12 months of storage.

## 4. Conclusions

The different processes during eel canning led to changes in the parameters reflecting the oxidation and in the antioxidant properties, both in the eel muscle and in the filling oils. However, canned eels packed in sunflower, olive, or spiced olive oils presented acceptable values for the parameters indicating fat oxidation after 12 months of storage.

Peroxide and TBARS values were determined by the filling medium and the storage time. At the end of storage, the peroxide indices were similar in the three types of preserves, while the TBARS values were higher in the preserves in olive oil.

Canning involves high heat treatments during precooking and sterilization steps, which promote changes in the fish. The fish quality after processing and storage is a result of a combination of the phytonutrient extractability, loss of compounds by degradation and leaching, and interaction between the fish and the filling medium. Canning caused the exchange of different components between the eel muscle and the filling oil used, increasing antioxidant capacity and vitamin E content in eel muscle after canning. The magnitude of these exchanges was dependent on the filling medium.

The antioxidant capacity of fresh eel is very low, but it increased significantly in canned food due to the filling oil. After one year of storage, canned eels packed in sunflower oil and the sunflower oil used as filling medium presented higher antioxidant capacity and vitamin E content than canned eels packed in olive oil or spiced olive oil and their respective filling oils. However, canned eels packed in olive oil or spiced olive oil and their filling oils presented higher total phenol contents.

The canning process and subsequent storage preserved a great part of the antioxidant capacity and vitamin E content of the filling medium, which is of interest to the consumer. All oils used as filling media (sunflower oil, olive oil, and spiced olive oil) are of great nutritional interest, and they could be used in canned eels. Therefore, the decision on the oil to be used must be based mainly on its availability and price, and the market to which the product may be destined.

## Figures and Tables

**Table 1:** The inhibition at 50% (IC_50_; mg/mL) values of European eel and filling oils after each step during the canning process and after 2 and 12 months of subsequent storage.

Steps	Eel Muscle		Oils	
Raw	---	107.05 ± 9.63 ^a^	Sunflower oil	7.88 ± 0.17 ^a^
			Olive oil	13.09 ± 1.08 ^b^
Frying	In sunflower oil	25.24 ± 5.39 ^b^	Sunflower oil	7.29 ± 0.08 ^a^
	In olive oil	28.39 ± 4.94 ^b,d^	Olive oil	13.96 ± 1.07 ^b^
Sterilization	In sunflower oil	36.43 ± 4.32 ^c,e^	Sunflower oil	7.51 ± 0.48 ^a^
	In olive oil	34.71 ± 1.42 ^c,d^	Olive oil	12.88 ± 0.18 ^b^
	In spiced olive oil	30.28 ± 1.77 ^c,d^	Spiced olive oil	16.85 ± 0.62 ^c^
2 months storage	In sunflower oil	31.77 ± 2.83 ^c,d^	Sunflower oil	8.57 ± 0.48 ^d^
	In olive oil	38.31 ± 3.32 ^c,e^	Olive oil	12.58 ± 0.44 ^b^
	In spiced olive oil	35.03 ± 6.02 ^c,e^	Spiced olive oil	16.11 ± 0.28 ^c,f^
12 months storage	In sunflower oil	36.77 ± 5.44 ^c,e^	Sunflower oil	10.34 ± 0.24 ^e^
	In olive oil	40.67 ± 0.53 ^e,f^	Olive oil	15.75 ± 0.67 ^f^
	In spiced olive oil	43.54 ± 1.59 ^f^	Spiced olive oil	15.92 ± 0.26 ^f^

Mean values of at least three determinations ± standard deviation. ^a–f^ Mean with different superscripts in the same column were significantly different (*p* < 0.05); IC_50_: extract concentration (mg/mL) required to decrease the initial 2,2-diphenyl-1-picryl-hydrazyl-hydrate (DPPH) concentration by 50%.

## Data Availability

Not applicable.

## Supplementary Materials

The following are available online at https://www.mdpi.com/article/10.3390/foods10040790/s1, Figure S1: DPPH radical scavenging activity of different extracts (100, 50 and 12 mg/mL) of muscle eels packed in sunflower (**A**), olive (**B**) and spiced olive (**C**) oils after each stage of processing and storage., Figure S2: DPPH radical scavenging activity of different extracts (25, 10 and 5 mg/mL) of the filling media after each stage of canning process and subsequent storage. (**A**) sunflower oil; (**B**) olive oil; (**C**) spiced olive oil.
