# Supplementary material for: Oxidative Stability and Antioxidant Activity in Canned Eels: Effect of Processing and Filling Medium
Source: Foods. 2021 Apr 7;10(4):790. doi: 10.3390/foods10040790 (PMC8067596; doi:10.3390/foods10040790)
Supplement: Supplementary file 1 [file foods-10-00790-s001.pdf]

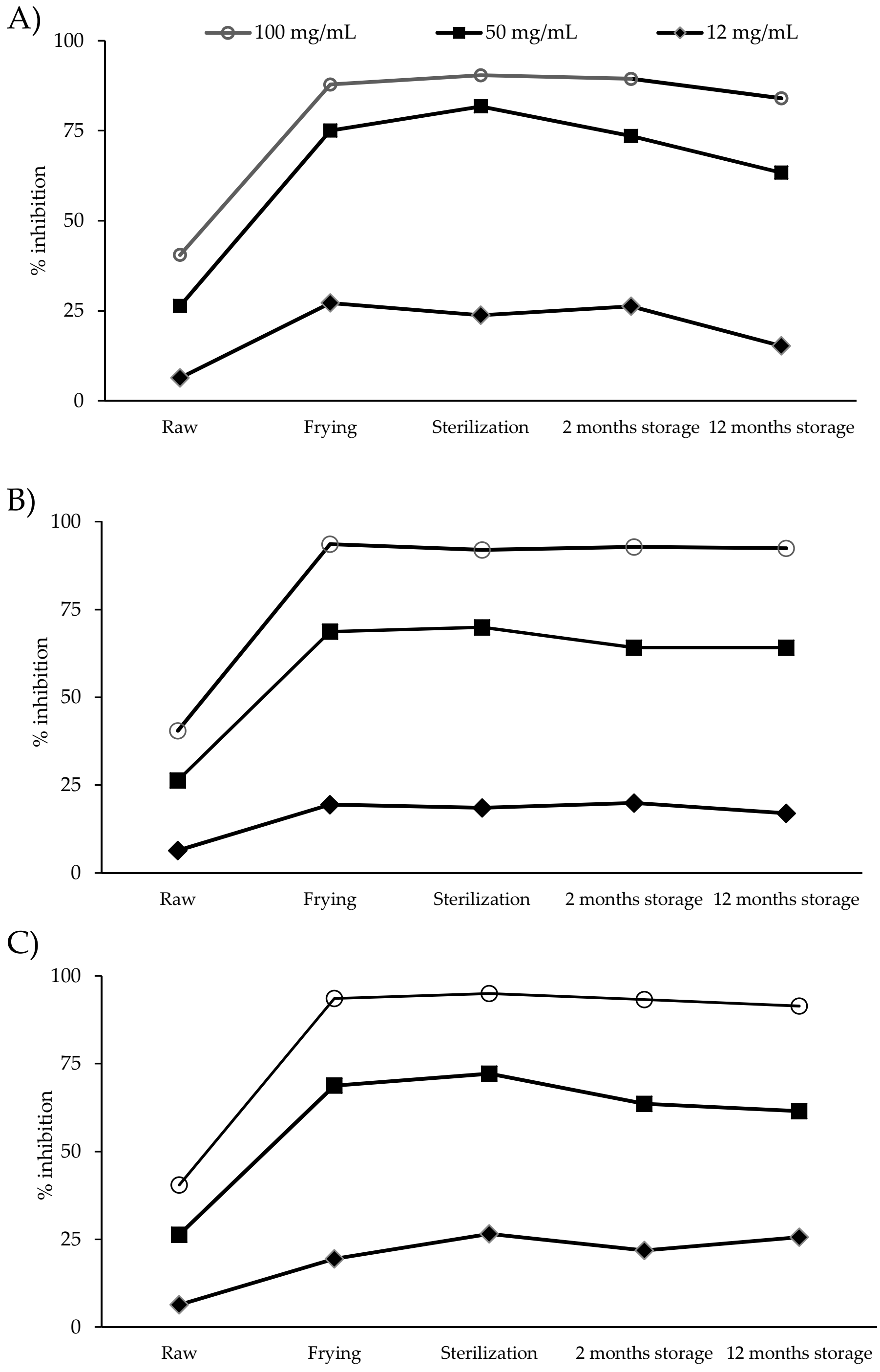

Figure S1. DPPH radical scavenging activity of different extracts (100, 50 and 12 mg/mL) of muscle eels packed in sunflower (**A**), olive (**B**) and spiced olive (**C**) oils after each stage of processing and storage.

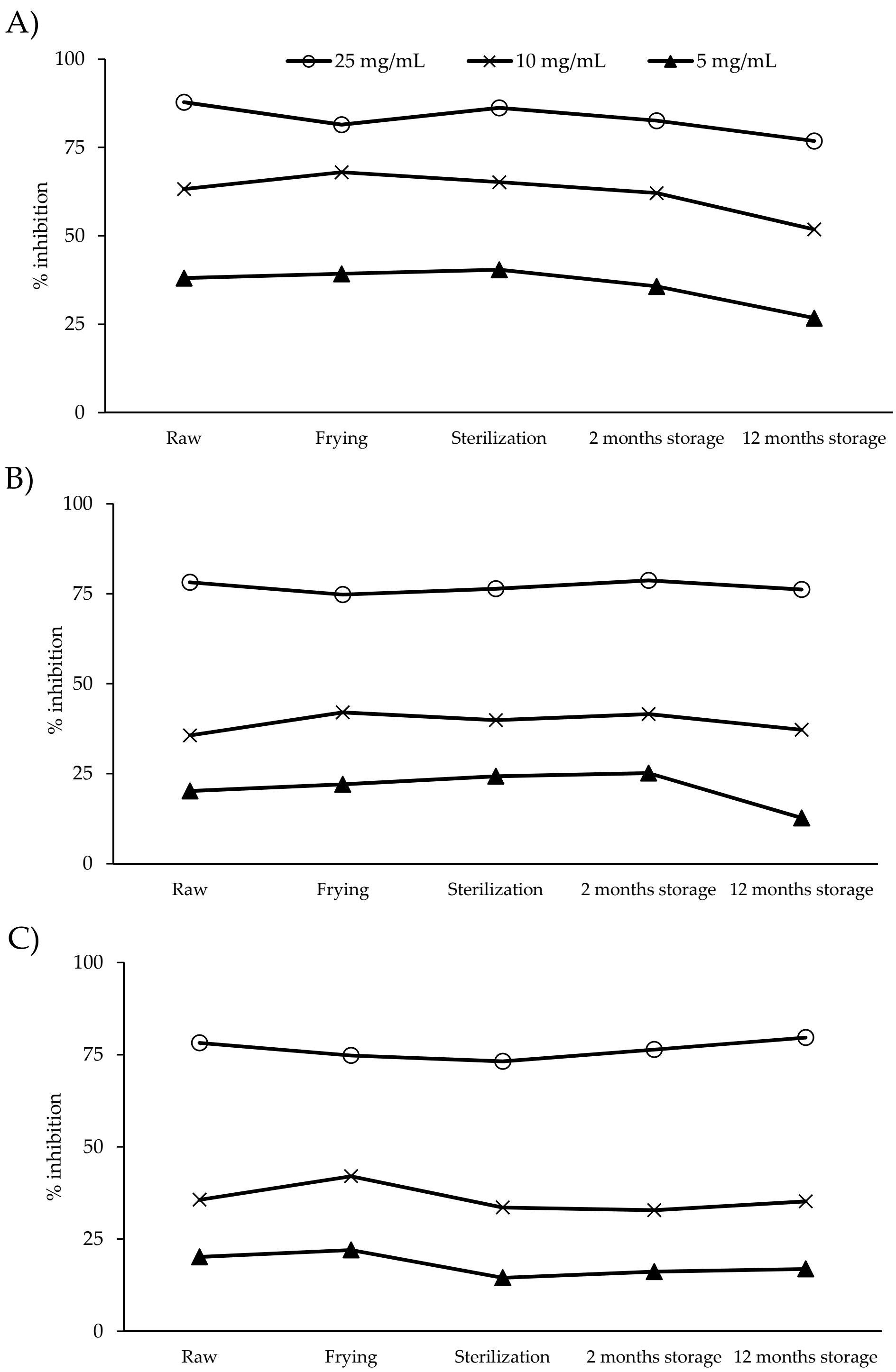

Figure S2. DPPH radical scavenging activity of different extracts (25, 10 and 5 mg/mL) of the filling media after each stage of canning process and subsequent storage. (**A)** sunflower oil; (**B**) olive oil; (**C**) spiced olive oil.
